# Multiuser design of an architecture for social robots in education: teachers, students, and researchers perspectives

**DOI:** 10.3389/frobt.2024.1409671

**Published:** 2024-12-16

**Authors:** Daniel C. Tozadore, Roseli A. F. Romero

**Affiliations:** ^1^ CHILI Lab, School of Information and Computer Science, École Polytechnique Fédérale de Lausanne (EPFL), Lausanne, Switzerland; ^2^ Robot Learning Laboratory, Instituto de Ciências Matemáticas e de Computação (ICMC), University of São Paulo (USP), SãoCarlos, Brazil

**Keywords:** social robots, education, HRI, teachers, children–robot interaction, interactive designn

## Abstract

Research on social assistive robots in education faces many challenges that extend beyond technical issues. On one hand, hardware and software limitations, such as algorithm accuracy in real-world applications, render this approach difficult for daily use. On the other hand, there are human factors that need addressing as well, such as student motivations and expectations toward the robot, teachers’ time management and lack of knowledge to deal with such technologies, and effective communication between experimenters and stakeholders. In this paper, we present a complete evaluation of the design process for a robotic architecture targeting teachers, students, and researchers. The contribution of this work is three-fold: (i) we first present a high-level assessment of the studies conducted with students and teachers that allowed us to build the final version of the architecture’s module and its graphical interface; (ii) we present the R-CASTLE architecture from a technical perspective and its implications for developers and researchers; and, finally, (iii) we validated the R-CASTLE architecture with an in-depth qualitative analysis with five new teachers. Findings suggest that teachers can intuitively import their daily activities into our architecture at first glance, even without prior contact with any social robot.

## 1 Introduction

Technology is being developed faster than ever (Roser, 2023). However, some of its outcomes are far from achieving their full potential. Socially assistive robotics (SAR), a field of robotics that focuses on assisting users through social rather than physical interaction, is a good example of this category of technology with many boundaries to be deeply extended (Matarić and Scassellati, 2016). In education, for instance, social robots for human–robot interaction (HRI) have significantly grown as a research field due to their advantages in enhancing students’ learning experience. Such advantages are simply momentarily boosting student motivation by being a novelty (Van Minkelen et al., 2020), being supportive coaches in language learning activities (Konijn et al., 2022), providing a tangible and tutor peer for studying (Ligthart et al., 2023), stimulating the feeling of rapport building and responsibility of taking care (Kory-Westlund and Breazeal, 2019), an indefatigable companion for wellbeing exercises in schools (Scoglio et al., 2019), and consequently, but not always, leading to learning gains (Johal et al., 2018).

Two noteworthy gaps are still underexplored in this field: the difficulty of conducting long-term studies, which has resulted in a significant decrease in the number of experiments lasting longer than two sessions (Woo et al., 2021), and the limited number of studies involving SAR in education that incorporate teachers and stakeholders in their field studies (Smakman et al., 2021a). These phenomena are often attributed, in addition to the many technical challenges, to the highly demanding multidisciplinary level that using SAR in education requires (Belpaeme et al., 2018) and to the teachers’ lack of time due to their already overwhelming workload (van Ewijk et al., 2020). As a result, few studies using social robots are published in journals, and this number is even smaller when it comes to long-term interaction studies (Woo et al., 2021).

Additionally, the success of long-term experiments, from the children’s motivation perspective, depends on mitigating the monotony inherent in the repetitive behaviours of robots once the initial novelty has worn off (de Jong et al., 2022). Studies have tackled this issue by incorporating adaptive behaviours into the robots, aiming for personalised treats for each child (Alam, 2022). These aspects are not only important to keep children motivated for longer periods of time but also to align with the principles of Industry 5.0, where human-based approaches are the core of the next industrial revolution (Ordieres-Meré et al., 2023). However, there is a lack of scalability in their approach to other activities and contents, as well as no deep accounting for the role of teachers in their implementation.

Concerning teachers’ incorporation into the design process and their willingness to adopt social robots, much cooperation and teamwork building has yet to be done between teachers and researchers. Although it is unfeasible to miraculously extend teachers’ daily hours beyond the conventional 24-hour limit, collaborative efforts can be directed toward the development of algorithms capable of automating certain aspects of their workload. This technological intervention can potentially alleviate some of the burden on teachers, thereby contributing to time savings in their professional responsibilities. For example, teachers could use robots to perform fixation exercises after classes and correct examinations with autonomous assessment algorithms (Lin et al., 2020).

One key element for achieving this desirable synergy is efficient communication between the involved parts (Arnold et al., 2021). Therefore, we postulated that graphical interfaces where teachers can insert their content through tables and see charts of student performance can foster the discussion of design, implementation, and result analysis, which are essential for bringing teachers into the loop in such studies (Szafir and Szafir, 2021).

Taking into consideration the mentioned advantages, limitations, and opportunities, the R-CASTLE (Robotic-Cognitive Adaptive System for Teaching and Learning) project emerged, aiming to deliver a unified HRI setup for experimental studies in education with a set of goals based on literature and practical needs: (1) providing the students with a tutor robot for personalised adaptive learning through multimodal analysis (autonomous content approaching in fixation exercises); (2) facilitate teacher participation by affording easy content insertion, system variable setting, and visual analysis of student and system performance in the executed sessions; and (3) allow more practical participation of researchers through visual reports of algorithm performance analysis and easy method changing as the system’s functionalities.

In this work, we present the final state of the R-CASTLE architecture, the methodological approaches to address the limitations and achieve the goals, and the lessons learned throughout its studies and development. More concretely, we summarize the performed user studies, focussing on their outcomes and implications for the technical implementation that is presented next. Additionally, a user validation was performed with five teachers who had not participated in any activity with social robots before. Therefore, they delivered a first-time perception of our solution, fitting the conditions in which we expect our system to be applied. Qualitative analysis of this validation confirms our hypothesis that the R-CASTLE has a high potential to promote social robots in educational settings by fostering collaboration between teachers and researchers.

Therefore, the main contribution of this work is a high-level analysis of the multiple user studies conducted at every step of the project and their implications from the teachers’, students’, and researchers’ points of view. We also present an analysis of how these results and adjustments impact long-term studies and teacher–researcher communication. Due to COVID-19, the long-term validation of the architecture in classrooms was not possible, leading to a deeper evaluation of teacher perspectives regarding using robots in the classroom in their activities. The results of this qualitative analysis showed a higher coherence with the teachers’ statements about adjustments made after the user-centric studies during the architecture design stage. Teachers also suggested that the interfaces for easy translation of their activity to HRI setups, as well as for the evaluation of the performed activities, play a key role in the acceptance of adaptive systems by teachers in both commercial and research applications, despite the main challenge at the moment being the acceptance of the decision-making stakeholders of the school.

The qualitative analysis with the teachers at the end is an important contribution itself because, unlike many studies that only address the teacher perceptions of social robots, it also addresses technical details and the researchers’ intervention as variables to be considered in long-term studies.

The remainder of this paper is organised as follows. In Section 2, we present the literature that motivated this research. In Section 3, several studies performed with students and teachers are related to understanding how to design and implement an intuitive and autonomous architecture that can be effective for both of them. In Section 4, we show the final result of the designed interfaces for the architecture based on the studies from the previous section. Section 5 presents a qualitative analysis conducted with teachers who have never interacted with social robots before and their impressions of how this tool can foster communication among teachers and researchers for experiments using social robots. Finally, in Section 6, the conclusions and future work are presented.

## 2 Literature review

Teacher engagement is essential to the development of social robots in educational environments because teachers are more sensitive to practical and ethical concerns, and they know how to approach them with the students (Jones and Castellano, 2018). Although most of the stakeholders in education are aware of the advantages, risks, and implications of social robots (Smakman et al., 2021a), the main challenges concerning teachers and social robots cited in the literature are related to the potential additional workload, which is caused by two main points: time management and technical knowledge (Belpaeme and Tanaka, 2021; Sonderegger et al., 2022).

This phenomenon is not exclusive to HRI in education. It is, indeed, aligned with the barriers that most innovative technologies face to reach classrooms. For example, according to the teachers, some crucial factors hampering the adoption of AI in their activities are lack of time, lack of training, and unfamiliarity with new technologies as well (Kipouros, 2018). A survey of more than 2000 K-12 teachers from four countries (Canada, Singapore, the United Kingdom, and the United States) by Mckinsey & Company reported some critical problems of the current educational system that can be smoothed with efficient technological solutions (Jake et al., 2020). The study claims that areas with the biggest potential for automation are the preparation of activities, administration, evaluation, and feedback. Conversely, actual instruction, engagement, coaching, and advising are more immune to automation. The report also concludes that automation has great potential to save teachers’ time in repetitive tasks, so they can use this time for tasks in which teachers are directly engaging with students, such as behavioural-, social-, and emotional-skill development.

Social robots have the advantage of providing an embodiment device for deploying a practical use of this automation. However, they come with all these already discussed challenges combined with the consequences—mostly complications—of the hardware component (Youssef et al., 2023), not only in the mechanical part but also in the robots’ shape and behaviour. Teachers’ lack of knowledge about social robots can influence the results of research in which their opinions are taken into account, for instance, the difficulty teachers have in understanding and visualising the real scenarios of social robots’ applicability and, thus, providing feedback inconsistent with feasible solutions (van Ewijk et al., 2020). Furthermore, studies measuring teachers’ negative attitudes toward social robots showed that a lack of prior experience with robots was the strongest predictor of negative attitudes, suggesting that increased exposure to social robots in teacher education might be an effective way to improve educator attitudes toward robots (Xia and LeTendre, 2021).

Such implications have a high impact on the research done in educational settings. A literature review showed that, in the last decade, very few studies using social robots have reached out to real classrooms in long-term experiments (Woo et al., 2021). By analysing other studies, this review concluded that most of the reasons are due to the difficulty in deploying autonomous robots in educational settings and the challenges of involving teachers in the loop. Consequently, research on social robots has not been widely published in educational journals compared to other technological methods.

In a qualitative study to further investigate the correlated issues, guidelines were proposed to approach these common difficulties (Ahmad et al., 2016). Among others, the main conclusions of the authors when interviewing eight teachers were: The robot should be able to answer repeatedly asked questions in the classroom; there is a need to design a dialogue-based adaptation mechanism to adapt to the children’s emotions and personality in real-time; robots keeping track of a child’s memory can in-turn motivate children more; and most importantly, designing easy-to-use interfaces for teachers to update new lessons for long-term engagement is key. Related to the last point, in this study, teachers emphasised the importance of their involvement to keep the robot engaged and involved during the learning process for a long time. To do so, it is important to design interfaces that allow them to manage the robots.

Based on the conclusions reported in the literature, it is evident that developing tools and methods to support and enhance teachers’ understanding, familiarisation, and use of social robots is crucial for the widespread adoption of this technology, both in research and commercial applications. Furthermore, few works addressing interfaces for social robots in education have presented an evaluation after putting the design process into operation in the classroom to obtain evaluations from the teachers’ point of view based on the developed tool. Although there has been a great interest in the topic in the last decades, resulting in many reports and qualitative studies on the topic, very few tools have been presented to approach the issue. Providing the advantages of social robots with adaptive behaviours to students and addressing the challenges of including teachers in their end-to-end design is one of the pain points of social robot research in education. This is the bridge that this project was aiming to build by providing a concrete tool resulting from many iterations of field studies.

## 3 User-centric studies

Throughout the years of the project, many studies were conducted to understand both student and teacher attitudes toward different systems’ functionality. The study outcomes combined with feedback used to guide the module development are presented in the next section. Such studies are important for understanding user preferences because their perceptions depend on different factors, such as culture and technology familiarisation (Kanero et al., 2018; Lim et al., 2021). Therefore, in the current section, the studies previously conducted and their importance to the project are summarised, including the lessons learned and how they were taken into consideration in the final version of the system. It is important to note that, although they have already been published, a deeper analysis of the complete series of studies and how they shaped each component of the architecture is still needed.

### 3.1 Student expectations toward robot behaviours

First, it was important to understand the aspects that students expect to have present in the robot in the specific context that this system as designed to be used because those expectations may vary according to culture (Šabanović, 2010; Chi et al., 2023). Thus, we deployed a sequence of studies to evaluate these factors, such as the analysis of variation of the robot, as outlined in Table 1 ^1^. Results from these studies helped to understand the importance of building an autonomous robot that can interact with students through dialogue and vision recognition. Hence, we implemented these systems as explained in Subsections 4.2 and 4.3 for the dialogue and vision systems, respectively. In addition, the findings pointed out that the regional context strongly aligns with results from the literature.

**TABLE 1:** Summary of studies with students.

Study	Key findings
Robot’s behaviour impacts (Tozadore et al., 2016)	• Robot’s interactivity significantly impacts participant learning and enjoyment • High interactivity leads to higher quality interactions • Repetitive behaviours can decrease children’s motivation
Gamification (Pinto et al., 2015)	• Implementing gamification improves participant performance and engagement • Visible cues (e.g., LED colours) and body language compensate for lack of facial expressions in the robot
Robot autonomy (Tozadore et al., 2017)	• Participant perceptions can change based on whether the robot is teleoperated or autonomous • Teachers find teleoperation impractical for regular use • Autonomous robots are perceived as more intelligent by students

### 3.2 Co-designing with teachers and end-to-end initial experiments

After drafting the needs of the students, we focused on the main stakeholders in educational contexts: the teachers. In this phase, we showed a group of teachers the outcomes from the experiments with the studies and the initial implementation of the modules in their initial stage to collect their feedback. Afterwards, we performed a two-cycle end-to-end experiment with teachers and students. The results are condensed in Table 2.

**TABLE 2:** Summary of studies focussing on the teachers.

Study	Key findings
Formative workshop and co-design with teachers (Tozadore and Romero, 2020)	• Opening the platform for teacher use requires addressing resistance due to the time and effort required for learning • Invited 14 teachers to a workshop, explaining social robots, their capabilities, and challenges, receiving feedback • Teachers found the system complex initially but felt more willing to adopt after the workshop • Teachers emphasised making system functionalities similar to technologies they already use • Two teachers agreed to further implement the system with their students
End-to-end experiment with teachers and students (Tozadore et al., 2019a)	• Teachers implemented content into the R-CASTLE system for fixation exercises with students, underwent two development cycles • The First cycle involved data collection with low adaptive behaviour, focussing on Portuguese grammar content • System autonomously evaluated student answers using the Google text-to-speech and Edit-distance algorithms • Teachers analysed results and modified exercises for the second cycle based on the system and student limitations • The Second cycle featured personalised and fully adaptive robot behaviour, resulting in higher student perception of a robot’s intelligence

The workshop’s outcomes showed the importance of providing intuitive means for teachers to insert their content when participating in research works. Therefore, we implemented the interfaces of the dialogue (Subsection 4.2) and vision module (Subsection 4.3) to allow customisation. We also drafted the content (Subsection 4.5) and the evaluation (Subsection 4.7) module interfaces.

Regarding personalisation in long-term interventions in children–robot interaction, deploying the system with the students in multiple sessions made two facts evident: adaptation and personalisation are key to student motivation for the next session, and a good strategy to provide the same effect is slowly presenting the robot’s complete interaction repertoire. For instance, when children learn that a humanoid robot can dance, they want to see it immediately, using all its limbs, lights, and speakers. We observed that by making the robot dance using one new resource every time in the next section, it became a novelty, extending the “surprise” elements of the robot to future meetings.

## 4 Architecture modules and graphical interfaces

Programming a robotic system to achieve cognition involves integrating multiple components that mimic human cognitive processes, such as perception, learning, reasoning, and decision-making (Cangelosi and Asada, 2022). The system must process sensory data through tools like computer vision and speech recognition, represent knowledge through structured formats, and adapt by using machine learning techniques. The system needs logical reasoning to solve problems, a memory structure for storing information, and attention mechanisms to focus on important stimuli. Finally, self-regulation and metacognition allow the system to monitor and adjust its own cognitive processes for autonomous decision-making and error correction. In this section, we present how we programmed our solution to afford cognitive skills based on the feedback received during the interaction design studies presented in Section 3.

**FIGURE 1:**
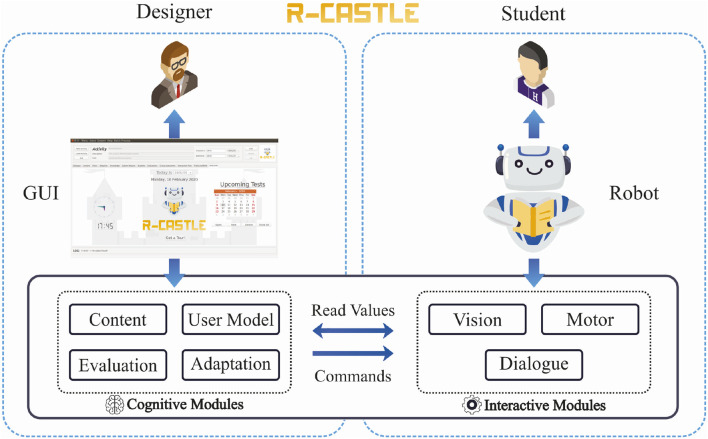
R-CASTLE high-level scheme: how each type of user interacts with the R-CASTLE system. Designers can manage operational and content settings through the GUI, while students interact with the system through a social robot connected to the architecture.

**FIGURE 2:**
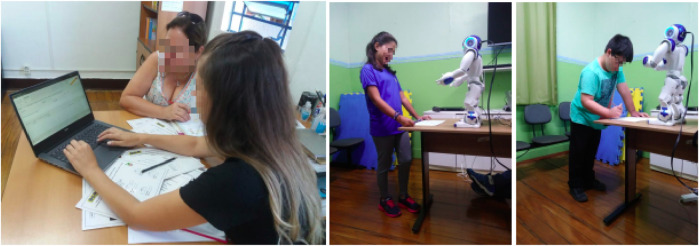
Users of the architecture: teachers inserting content in the interface (left) and students interacting with the robot in the exercise sessions (middle and right).

A high-level scheme of the resulting architecture is illustrated in Figure 1. Its implementation was based on student, teacher, and researcher feedback, allowing an easier and more intuitive usage of a cognitive system that can adapt to student performance and provide the designers (teachers or researchers) with adapted parameters. A video showing an end-to-end configuration and execution example of the architecture works is publicly available on YouTube^2^. Externally, the system is designed for use by teachers and students, as shown in Figure 2.

The figure illustrates that the designers interact with the system through the GUI. As mentioned before, designers can be teachers and researchers. Joint usage is also envisioned, for instance, when planning new activities or discussing study results. The GUI allows them to insert content, set parameters, manipulate data, and evaluate/visualise the results of previous sessions. Students will interact with the system through a social robot connected to the architecture.

Although we used an NAO robot in these studies, other robots that afford social simulation capabilities can be connected to our architecture through the robot operating system (ROS) by implementing publishers and subscribers to communicate the robot’s resources (text-to-speech, speech-to-text, and engine connections), as better detailed in Pinto et al. (2018).

The *Interactive Modules* (bottom right in Figure 1) were the first ones developed to allow the system to be autonomous. They are the Vision module, responsible for handling the information exchanged via the robot’s cameras; the Dialogue module, responsible for the audio information needed during the interaction; and the Motor module, responsible for connecting different output devices and moving the engines of the connected device. The technical functionalities of these modules were presented in previous work (Tozadore et al., 2019b). In the following subsections, we present how these functionalities were linked to the corresponding graphical elements in the architecture’s final version.

Finally, the *Cognitive Modules* (bottom left in Figure 1) are the modules developed to afford short and robot long-term adaptation for each student. They are “cognitive” because they have functionalities to process and store data to generate knowledge about the students, contents, and decision-making processes.

The inputs to R-CASTLE come from interactive modules containing information about students and from the designer, specifying the hyperparameters of the system. Thanks to the inputs, the system can adapt itself according to the adaptation function (Equation 1) to attend to the student’s necessities during the interactions, such as getting more attention from the student based on face deviation and emotion recognition and changing the level of the learning process. The R-CASTLE output consists of the robot’s adapted behaviour and auto-generated reports of student performance. Regarding behaviour adaptation, the robot can use more difficult or easier questions or perform more dialogues regarding students’ personal preferences if the system realises signs of disengagement (multiple face deviations, face display of confusion, long times to answer). Due to the growing concern about data privacy and especially because the studies performed are research studies, the database was implemented to be only locally managed to guarantee data privacy without a connection to the internet.

For a more practical understanding of its application, let us take the scenario reported by Tozadore et al. (2019a) as an example, where students interacted with the robot twice to learn about environmental waste. The robot used its sensors to capture student answers and behaviours through image processing and speech recognition (interactive modules), utilised real-time information to adapt its behaviour and adjust question difficulty for each student (adaptive module), and collected data on students’ personal preferences, such as music, food, and sports (User Model module). During the second interaction, the robot leveraged personal and performance data for each student (stored in the User Model and Evaluation modules, respectively) combined with the content provided by teachers (stored in the content module) to enable personalised interactions. As in the first interaction, the robot continuously monitored student responses and displayed behaviours, using the adaptation module to decide whether to trigger personal conversations to regain attention or adjust the difficulty of the questions to offer an appropriate challenge level. As a result, students reported higher self-perception of learning and rated the robot as more intelligent in the second session. The system evaluated student performance in both sessions and generated graphical reports for the teachers (evaluation module), who discussed the results with the researchers. Teacher programming of the content and analysing the student outcomes and student interactions with the system are shown in Figure 2.

The next subsections present the delivered user interface after the studies presented in Section 3.

### 4.1 GUI

**FIGURE 3:**
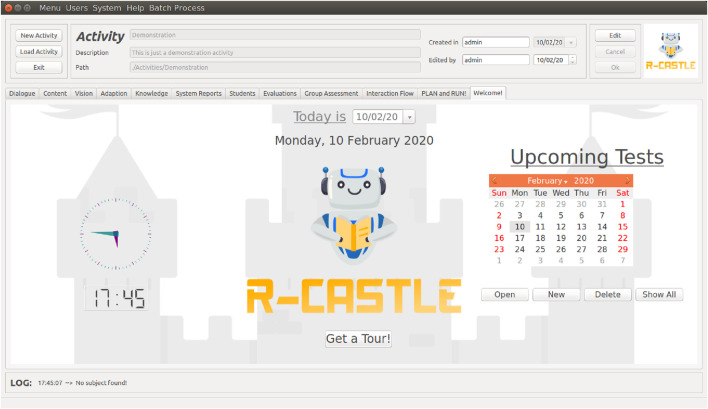
Welcome window of the R-CASTLE architecture. The upper part with the activities and module tab is fixed, while the bottom part (with the castle image on the background) changes according to the selected module.

The designed window is the Welcome screen, displayed in Figure 3. It was developed in PyQT5^3^ to easily integrate the other algorithms, and it was also developed in Python. From a user’s view perspective, the window has a fixed top bar that contains the activity summary and the buttons to manipulate activities, and the middle component, with the clock, logo, and calendar, is the *tab widget*. This widget is dynamic, and changes to the tabs corresponding to each module explained in the next subsections. Therefore, all the screenshots displayed next are widgets that will only change in part of the interface.

### 4.2 Dialogue

**FIGURE 4:**
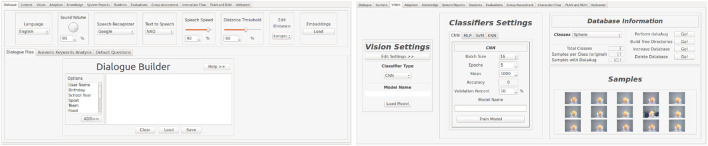
Dialogue module tab (left picture): Designers can set the parameters, build personalised dialogue, and set keywords for positive, negative, and dubious student answers. Vision module tab (right picture). Settings of the type of classification algorithm, models, and dataset are available through this module’s interface.

The Dialogue tab, shown in the left image of Figure 4, allows designers to set the parameters responsible for keeping the conversations flowing autonomously. Speech recognition and text-to-speech are allowed through Python *SpeechRecognition* ^4^) library and *Softbank Text-to-Speech API* ^5^). If any NAO robot is available, the text-to-speech from Aldebaran switches to the *Google Text-to-Speech* (GTTS) library^6^. Designers can configure several sound settings through the dialogue window in the GUI, such as volume, speech speed, algorithms for matching the users’ answers with the expected ones, and a similarity threshold to match as a correct answer. Robot speeches can also be built in the dialogue tab. They could be any other information the system wants to exchange that is not related to the content. User interests can be allowed along with the utterance, for example, talking about the preferred music of the current user. The speeches are saved in files and later chosen based on which part of the interaction they will appear in the Interaction Flow tab. It also takes into account the keywords that the designer inserts: affirmation, negation, and doubt. The keywords affirmation and negation are used in cases in which the system asks a regular question to the students and expects a binary answer of yes or no. Then, all the words filled in the corresponding group will fit. Keywords of doubt are analysed in every user’s answers. If a high frequency of these words is detected, the system can start to repeat the questions, lower the speech speed, or send a message to the adaptive system to lower the difficulty of the questions. The sentence-matching algorithm evaluates the correctness level of the user’s answers based on the expected answers registered by the designers. To date, no large language models (LLMs) have been trained or implemented in the architecture; this is a potential follow-up point.

### 4.3 Vision

As a primary responsibility, the vision system manages the device’s camera and recognition algorithms. Second, it also sends information to the modules that will process this information. Images of user faces, for example, are sent to the User Model system, whereas the information on the users’ facial expressions, such as emotion and face gaze, are used by the Adaptation module.

For the image recognition in the tasks, designers can use the Vision module tab, as shown in the right image of Figure 4, to choose which one will be used in the next session and change their parameters before training in the algorithm settings section. Machine learning methods are available, such as multi-layer perceptron (MLP) networks, K-nearest neighbour (KNN), support vector machines (SVM), and convolutional neural networks (CNNs). More details about the advantages and setbacks of using these methods can be found in Tozadore and Romero (2017).

The system uses the Python face recognition module for user recognition.^7^ The Haar Cascade Viola and Jones (2001) algorithm, implemented in the *OpenCV* library, is used for face gaze detection, as described in (Tozadore et al., 2019b). Finally, emotion recognition through facial expression is the result of a study where seven emotional states of Ekman’s model, such as *happiness*, *anger*, *sadness*, *fear*, *disgust*, and *surprise*, plus the *neutral* emotion, were trained to be detected by a CNN (Tozadore D. et al., 2018).

### 4.4 Adaptive

The Adaptive module aims to change the robot’s behaviour and the difficulty level of the approaching content based on the observed student indicators, which are expressed by their body language and verbal answers.

These indicators were divided into three major user skills. They are Attention (α) , Communication (β) , and Learning (γ) , with the corresponding indicators for each category: Face gaze (Fg) is used for Attention (as suggested by Abdelrahman et al. (2022)); Number of spoken words (nW) and User emotions (Em) are used for Communication (inspired from Shi et al. (2015)); and Time to answer (Tta) and Right/Wrong answer (RWa) are used for the Learning group (as in Lee and Jia (2014)). The average of the objective measures of each group results in a final major value of the class, named α to Attention, β to Communication, and γ to Learning, as shown in Table 3. For further details about the adaptation function and its foundations, please refer to Tozadore et al. (2019b).

**TABLE 3:** Objective measures by group.

Attention (α)	Communication (β)	Learning (γ)
Face gaze (Fg)	Number of words (nW)	Right/wrong answers (RWa)
Posture (p)	Emotions (Em)	Time to answer (Tta)

In this way, the abstract resulting functions with the corresponding vectors are represented as α=(Eg,P) , β=(nW,Em) , and γ=(RWa,Tta) , where the adaptive methods will combine these measures to predict a change in the robot’s behaviour according to the teacher’s definition. For instance, to be more or less interactive, to present more difficult or easier questions to students, to provide more or less help toward interventions, or to talk more or less about students’ personal preferences between tasks. Hence, Equation 1 shows a generalisation of the adaptive function regarding the inputs from Attention, Communication, and Learning.

$$F_{\mathrm{Adp}}\left(t\right)=\left(w_{\alpha }*\alpha \left(t\right)+w_{\beta }*\beta \left(t\right)+w_{\gamma }*\gamma \left(t\right)\right),t\in N.$$ (1)

The function outputs will have two utilisations: be saved and sent to the Assessment Module to produce the reports (to the designers about the interactions with the users) and to be used as input for the adaptive behaviour function.

**FIGURE 5:**
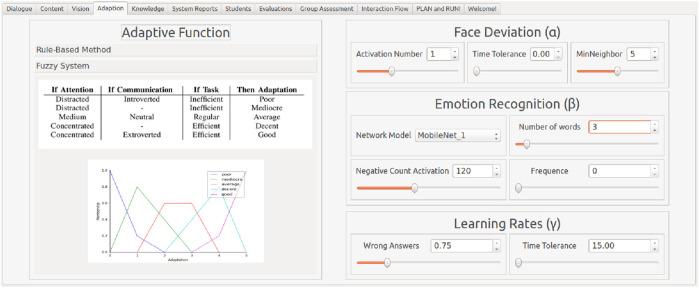
Adaptive module tab. Designers can choose between the methods on the left part and set their parameters on the right side of the screen.

Regarding the adaptation algorithms, there are three implemented methods for the adaptation calculation: rule-based (Tozadore D. C. et al., 2018), fuzzy systems (Tozadore and Romero, 2021), or supervised machine learning algorithms. Whereas the first two methods required parametrisation inputs of the designer and should be input through the interface, machine learning techniques should be hardcoded due to the expertise required in data handling and code development. In the interface of the adaptation module, the designers can set the parameters and the methods on the fly according to each activity, as shown in Figure 5.

### 4.5 Content

The GUI makes content insertion easy for teachers. The content is stored in the system database, and it can be approached in any late activity.

**FIGURE 6:**
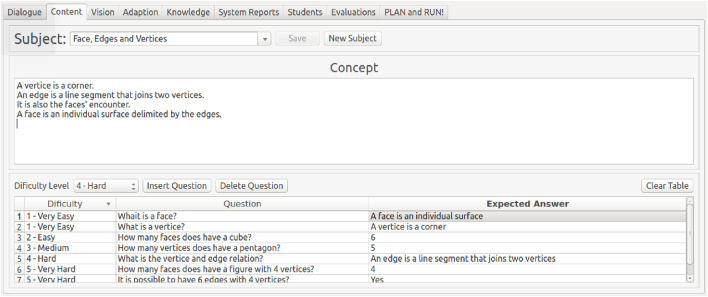
Content module tab. Designers can set all the topics they want to approach. The explanation of the topic goes in the “Concept” field, and questions are inserted in the table below it with their respective expected answers and difficulty levels.

The content is approached in topics (Subjects, in Figure 6). Each topic is an entry in the Content module that has a concept (an explanation of the topic from which the questions will be derived) and many questions.

For instance, if the activity is about animals, one can create a topic for each class, such as mammals, fishes, insects, and so on, and their concept would be the features that make them belong to this class, followed by their corresponding questions. An activity can have as many topics as needed and as many questions as needed. The designer should fill the concept text box, which is the utterance the robot speaks before starting the questions regarding the current topic. After that, the designer should insert at least one question of each level of difficulty followed by the corresponding expected answer. The pedagogic model of this proposal—the interaction of the student and the system conducted by the robot during the content approach—works like a quiz mode. It means that in each step, the robot gives explanations and asks questions about a topic that was inserted in the content module by the teachers.

Once the system has the capabilities of both speech and image recognition, the expected answers can be a sentence or an image. In the case of a sentence, the system analyses the answer by the dialogue system, as shown in Subsection 4.2. On the other hand, the answers that are expected as images are classified by the Vision module, as shown in Subsection 4.3 and also in Tozadore and Romero (2017). For instance, in the question “What is the 3D geometric figure that has no vertices and edges?” the answer could be verbally accepted as “A ball” or by reading the camera’s image in which the student would be showing a ball (as long as the designer set the question to be answered in this way).

### 4.6 User model

**FIGURE 7:**
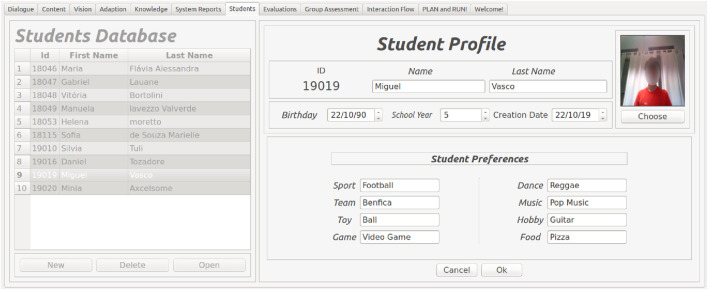
User database interface. The system stores users’ personal information (if needed) and topics to discuss, such as favourite music and food.

The User Model, or Student Model module, stores information about every student who interacted with the robot. The information includes their first name, family name, age, school year, birthday, user pictures, and eight user interests in sports, dance, teams, music, toys, hobbies, games, and food. This data can be autonomously collected or manually inserted through the user interface tab, as shown in Figure 7. All the data are stored in the user database, and the definitions of these interests are stored in the system’s knowledge database. Designers can perform transactions in the database manually in the corresponding window. Student interests can be used in small talk at the time that it was previously set or autonomously when a high frequency of bad readings (a high no-attention level or a high number of wrong answers) is detected by the system. This module’s features were highlighted in experiments within this project, as well as pointed out in other works in literature (Pashevich, 2022). More details about this module can be seen in Tozadore D. C. et al. (2018).

### 4.7 Evaluation

The Evaluation module reports the performances of both the system and students to the designers. For each activity, the teacher can access different graphs about the system assessment of each student or the whole class report. Another functionality is that the system can report on the performance of R-CASTLE in terms of the machine learning algorithms’ accuracy based on manual validation.

**FIGURE 8:**
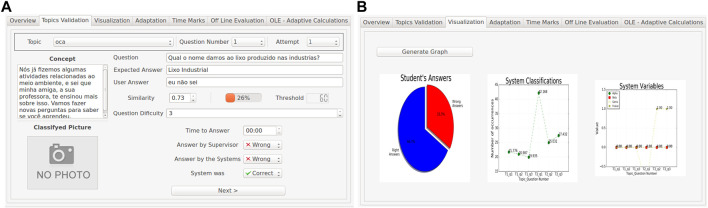
Validation **(A)** and visualisation **(B)** window of the evaluation module. Designers have to validate the outcomes of the autonomous algorithm (left picture) and the system and student’s performance are available for checking (right picture).

The evaluation module first presents an index of all the previously performed activities (Figure 8A). After selecting a session, it presents a human validation tab (Figure 8B), which allows designers to correct potential mistakes performed by the system’s autonomous evaluation.

The Evaluations tab shows the session’s information about the last sessions performed. It displays the user recognised by the system in this session, the configurations that the session was run, the user accuracy assessed by the system, the time the session started and ended, the designer, the robot or interactive device used, and extra observations that the person who executed the activities wanted to add. Another highlighted feature of the Evaluation module is the human validation of each system classification of the students’ answers through the GUI. This validation checks the performance of R-CASTLE’s algorithms in classifying the answers. After validating all the questions, graphs of both student and algorithm performances are shown. User performance is related to how many correct answers they gave based on the expected ones, while the performance of R-CASTLE is evaluated based on how many answers it classified correctly based on the human validation after the sessions.

The measures available for showing in these graphs are the observed values from the users; their corresponding skill values of *Attention* (α) , *Communication* (β) , and *Learning* (γ) ; the user performance according to the system’s evaluation, further according to human validation; the system performance in evaluating the user’s answers, further pursuing the best adaptation.

**FIGURE 9:**
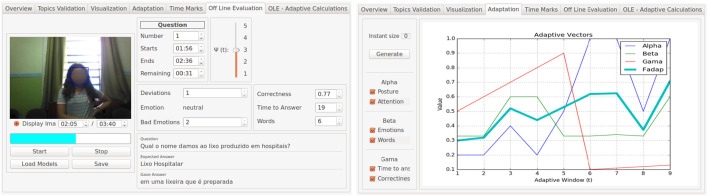
Off-line evaluation (OLE) tabs. Designers can use recorded videos to validate new parameters or completely new algorithms (left picture), and the outcomes of the new performance are shown in the graph (right picture).

Recorded videos made by the frontal camera of the robot are available to be watched later and used to retrain the classification algorithms. They are accessible in the “Time Mark” sub-tab of each evaluation. Every video has the time mark of the beginning of each question, and the person watching can jump to these specific moments. With the videos and the verbal answers stored, the sessions are available for as many reassessments as wanted in the tab “Off-Line Evaluation.” In this way, all the algorithms of students’ readings and classification can be evaluated with different parameters. The result of each algorithm configuration with the new parameters, weights, and tolerances are stored and available to be used in the adaptation methods at any time in the “Off-Line Evaluation (OLE) — Adaptive” tab (Figure 9). The results of new methods and configurations are shown in graphs immediately after the OLE end.

Therefore, beyond the functionality of keeping teachers aware of the student’s performance through evaluation graphics, the Evaluation module works as a laboratory to retest and maintain the machine learning methods at a high performance level. This module also fostered discussions between teachers and researchers regarding the outcomes of the studies performed using the architecture.

### 4.8 Researchers’ considerations and project limitations

The developed interfaces aimed to create an understandable layer between the software implemented and its interaction with the students through the robot. Although there are some windows that teachers do not feel comfortable using alone (for instance, the Vision module due to all the parameters to set), all the windows are important to promote explainability to them about how the system operates and to leverage the discussions about the experiments. Although the system was seen as a potential product to be later launched on the market, there is still a wide margin for improvement in the user experience part.

The fast in-loco configuration of new parameters for different activities and modularisation of the architecture allowed for rapid adjustment of the algorithms between studies and saved much researcher time. These features facilitated different studies to be modified and performed in a shorter term than the traditional operation of hardcoding. However, systematic tests are still required to validate how much time these approaches could spare from experimenters. They were planned to be performed at the end of this project but were aborted due to the COVID-19 pandemic. Therefore, the limitations of this project include the lack of more sessions for validation because final user studies did not take place due to the social distancing measures of the COVID-19 pandemic at the end of this project. A large-scale validation regarding how the proposed framework can benefit the researcher-teacher communication is still a topic worth further investigation with quantitative analysis because it was only done with a qualitative analysis, as presented in the next section (Section 5), for the same reasons as the studies were interrupted.

## 5 Usability test through qualitative analysis

To validate our proposal from the teachers’ perspective, we qualitatively analysed the data collected in interviews performed with five teachers who did not participate in the architecture development. The recruitment was done by sending an invitation to social media groups of teachers, and the first teachers to subscribe to the project were accepted if they fit the inclusion criteria. The inclusion criterion was teachers of elementary school who have more than 5 years of experience in classrooms, regardless of the use of technology they have in their classroom or their familiarisation with the topic. To preserve participants’ unbiased opinions, the final goal of the interviews (checking their perception of adaptive methods for social robots in the classroom) was not mentioned in the call for participation. Instead, the announcement only informed teachers that they would participate in a 60-minute conversation about technology in classrooms.

### 5.1 Participants

Registered participants^8^ were five teachers (named here T1 to T5) of elementary schools in different cities of the state of São Paulo, in Brazil, with an average age of 43.6 years (SD 9.39) and 24 (SD 8.86) years of experience in classrooms. To preserve their identities, we provide their profiles that can be useful to understand their opinion. The first participant (T1) was a retired teacher working with children approximately 6 years old in public schools only for more than 35 years until 2019. The second and fourth participants (T2 and T4) had similar profiles. They had only worked in the same private school, which both of them described as “Very motivated to adopt high-tech and innovative solutions for education.” Finally, the third and fifth participants (T3 and T5) were teachers who had worked in both private and public schools. They were asked to give their feedback based on both scenarios but to make their situation clear in their responses.

### 5.2 Methodology and structure

We used semi-structured interviews, a standardised method in which the experimenter has a set of pre-defined questions that guide the conversation toward key points the experimenter wants to investigate (Veling and McGinn, 2021). In the questions, we adapted versions of Perceived Ease of Use (Saari et al., 2022), Attitudes and Acceptance (Naneva et al., 2020), and Intention of Adoption (Louie et al., 2021) to be suitable for our research questions. Interviews were conducted via Zoom to facilitate the recording, transcription, and data analysis, as well as to support social distancing. Although most of the data were structured for objective measures, two researchers analysed the videos and the scripts, checking the conclusions we can draw from teacher opinions, following a simplified version of the work done by Ceha et al. (2022), where we used thematic analysis using the coding present in Appendix Table A1 in the Appendix. However, because we wanted to explore the different ideas to be used in creating activities with the interface, we did not provide any background scenarios to teachers. We rather asked them about their current activities and how they would adapt them to the interface.

We divided the interviews into three phases. In the first, we asked exploratory questions about the teacher’s profiles and opinions on social robots based on what they already know. In the second, we focused our investigation on social robots (in general) and their implications based on the definitions we set. Finally, in the third, we presented the R-CASTLE and performed a user validation with them. Throughout all the phases, we first asked for participant opinions in open questions and then triggered further focused discussions with alternative questions.

### 5.3 Results

#### 5.3.1 Exploratory phase

In the first phase, we asked several questions to understand the teachers’ *a priori* point of view about technology and social robots. First, we inquired about their familiarisation (or frequency of usage) with existing devices: smartphones, tablets, computers, robots, social robots, software with artificial intelligence, and other devices they would like to mention. The familiarisation was based on three criteria: Their personal use, the use of these devices as regular activities in their class time, and their use for extra-class activities/homework. A 5-point Likert scale was used for this question, where the items were: 0-“I’ve never used it/I don’t know anything about it”; 1-“I know what it is, but I never used”; 2-“I use it, but with a lot of difficulties that interfere [with] its utilisation; 3-“I use it, but with some difficulties that occasionally prevent me of doing what I wanted”; and finally, 4-“I use it and the difficulties I have very rarely prevent me from doing what I want.”

At this point, when asked about social robots, all the participants asked some form of “*What do you mean by social robots?*“ They were informed that they should follow the definition that came to mind and that we would come back at this point later.

**FIGURE 10:**
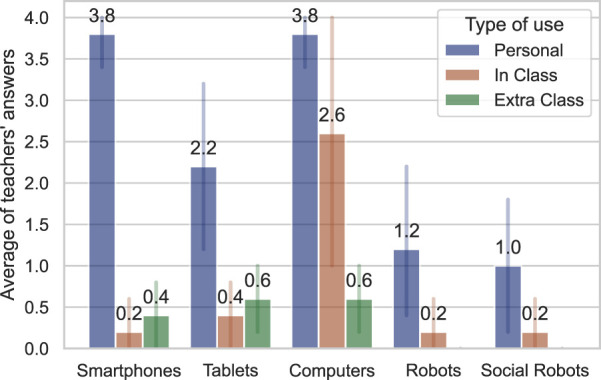
Teacher familiarity with different technologies.

The results of the teachers’ familiarisation are illustrated in Figure 10.

Results showed that teachers are very familiar with smartphones, computers, and tablets, using them very often for personal use and always as possible in their classes (in private school contexts, not in public ones).

The interview proceeded by asking what they thought about social robots when answering the questions and what they thought a social robot was.

Teacher opinions were convergent to some kind of personal assistant, like Alexa or Siri. What was the assumption that led T3 to answer that she uses social robots in her daily life and activities (fourth and fifth questions in Figure 10)? Only one teacher (T2) gave an answer related to physical embodiment, associating social robots with domestic cleaning robots. The other four teachers presented a concept relating the entity of social robots to personal assistants, where two teachers explicitly cited Amazon Alexa (they said they have it in their homes). In all these four later answers, the words “interaction” and “ human” were contained in their answer.

Not surprisingly, participants gave ideas about the robot’s use according to their definition, where T2 said they could be used to teach programming skills (assuming social robots are cleaning robots), and the others said the robot could assist teachers and students as a personal guide. Additionally, T1 added that the robot has the advantage of providing more media resources than already-used devices (tables and computers), and T4 said she thought this type of assistant could help her grade activities.

In contrast to research done previously on the popularisation of domestic robots (whether interactive or not), where teachers did not have a clear idea about robots and interactive devices (Serholt et al., 2014), the teachers gave definitions close to the one we are using in this survey.

We then asked: *Based on your conception, how do you envision their utilisation in your activities?* We asked them to think about their current activities and present examples whenever possible. Most teachers associated activities afforded by personal assistants, such as the robot being a personal tutor to each student (T1 and T4) and collecting data from the students autonomously for teacher evaluations (T3). T5 envisioned a different role for the robot, where it would also assist the teachers to prepare their content. T2, however, claimed that social robots (in her conception of cleaning robots) would not be useful in her activities at this point. Interestingly, all these features can be achieved using R-CASTLE, as discussed in Section 5.3.3.

#### 5.3.2 Teacher opinions after our definitions and explanations

Having finished the exploratory phase, which lasted approximately 10–15 min on average, we defined the term “social robots” and presented videos of social robots to teachers so the teachers could see the robots in action. We used the definition presented by Sunny et al. (2023): “*A social robot is an autonomous robot that can connect and communicate with humans and other social robots by adhering to the social behaviours and rules associated with its role in a group.*” Afterwards, we showed videos about social robots^9^.

Then, we asked questions to foster teacher opinions based on the definition we gave them, and they envisioned using these robots in their teaching activities with this new horizon. The result is presented in Table 4.

**TABLE 4:** Teachers' answers clustered by the adopted codes.

T#	Perceived advantage	Application in their activities
T1	Embodiment	Assistant for repetitive questions
T2	Storytelling, novelty factor, emotion, and personalisation	“Sharing circle”
T3	Generalisation and embodiment	Word bingo and animal mimicking
T4	Novelty factor, embodiment, and scalability	Dancing, singing, and exercising
T5	Generalisation and scalability	Vocabulary fixation

T2 corrected her understanding of social robots and found applications for social robots more quickly than the other participants. She was also amazed she could use them to spare her resources. *“Oh, I would love using them […], and I can find a lot of applications. As a teacher, I have always my voice very tired, and using them to sing or repeat some information would really help me physically.”*

An important observation was that every teacher commented on aspects of generalisation for the tasks and personalisation for the students. Whereas they realised that the robots have the potential to collaborate in diverse activities, they also realised the feature of affording personalisation to each student.

For the teachers in the context we expected regarding social robots and giving examples of their utilisation, we asked for the challenges and barriers they see for the social robots popularisation in open question. We clustered their answers according to the codes presented in Table 5.

**TABLE 5:** Teachers mentioned the main challenges according to the coding scheme.

T#	Perceived barrier
T1	Teacher familiarisation
T2	Access to the technology, price
T3	Teacher familiarisation
T4	Competitive attention with teachers
T5	Price, technical aspects, and teacher familiarisation

When asked if they were aware of scientific studies done in the field, all participants but T4 claimed they did not think there are many, especially in the Brazilian context. The reasons they mentioned were the same as reported in Table 5, and they added that research is not profitable for their institutions. Thus, it is not a primary interest of the schools.

On the other hand, T4 said, “*Yes, I think there is a lot of research going on because it is a growing area [social robots], but we are not noticed because, for the media, it is more interesting reporting other things*.”

At some point, all the interviewees mentioned the practical challenges that social robots face before becoming part of their regular teaching toolkit, such as the high financial cost (Pachidis et al., 2019), teachers’ lack of technological knowledge to deal with these robots (van Ewijk et al., 2020), how learning new methodologies, specially such a complex one, had an impact on their time management (Sonderegger et al., 2022), and how administrative layers of their school and children’s parents’ acceptance play a role in the adoption of social robots (Smakman et al., 2021b).

**FIGURE 11:**
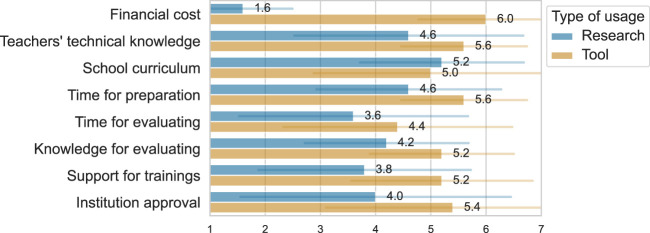
Teachers' opinions on a 7-point Likert scale regarding the barriers to using social robots in classrooms. In blue, considering they would participate in research, and in yellow, if they have to use it on their own as a regular tool.

Based on studies found in the literature, we brought together the main pain points that prevent social robots from being used as a regular tool in daily class activities. We presented these points to the participants and asked teachers to give weights to the challenges in two conditions: one considering they would participate in scientific study (research) and the other if they were to use this kind of technology as a regular tool for teaching. The results are shown in Figure 11 ^10^.

Participants gave more weight to the financial factor, assuming they were to use the robots as a regular tool, followed by the technological knowledge they assumed they would need and the extra time they would have to allocate for preparing the activities. The cost of using a robot as a research artefact would be less impactful, but the factors of not being part of the regular curriculum of the school and, again, teacher knowledge play a substantial role in this condition. For both conditions, the teachers gave less weight to the impact of evaluating student performance because they claimed it would be easy to simply transfer their regular evaluations to the new approach.

#### 5.3.3 R-CASTLE user validation

Finally, the discussion focused specifically on R-CASTLE. We started by showing the demo video (the same as in Section 4) and asking participants for their perception of the interface, which activities they thought they would be able to program using the interface, and the functionalities (windows) they thought they would use the most.

The responses highlighted various aspects such as motivation for children, adaptation to different learning styles, facilitating teachers’ work through automation, and enhancing interaction in the classroom. There was also a mention of the importance of adaptation for creating bonds, particularly in cases like autism, and the potential for robots to engage students who may not interact as much with peers or teachers. Overall, the responses suggest a recognition of the multifaceted impact that technology can have on education, from enhancing engagement to streamlining processes.

Regarding the challenges, all participants revealed that they still think it is more complex than they are used to; however, it was easier than they thought it would be. T2 and T4 (from the same school) said they are already using similar technology in their classes, a mathematics tutor that they had to load with different levels of content.

Diverse types of answers were given with regard to their preference for the functionalities. T1 claimed she would really like to explore all of them through their windows, T2 manifested high interest in the content insertion, T3 mentioned the evaluation, T4 voted for the studies scheduling system, and T5 liked the adaptation module.

Participants were requested to give examples of a daily activity they performed with their students to insert in the R-CASTLE. Without further prompting, they reported that they would be able to apply all the activities they mentioned before using R-CASTLE. When asked to choose one, T1 chose cooking classes, T2 chose vocabulary naming (bilingual classes), T3 chose fixation exercises of language, T4 said activities with autistic students (now that they are integrated into regular classrooms), and T5 said conversation circles.

Inspired by the evaluation we performed with teachers, which helped us to design the architecture, we asked teachers quantitative items about their perception of the architecture, in items regarding: (1) How easily do you think you could create your activities using this interface? (2) How easily do you think you could evaluate student performance with this system? (3) How do you judge the system’s potential as a product to be consolidated on the market? (4) How efficient do you think this methodology is for content addressing? (5) How long would you estimate it would take to familiarise yourself with this interface? (6) How efficient do you think this methodology is compared to the traditional one (that you already use)? (7) How much do you estimate your school’s intention to buy if it was available as a commercial product? (8) How much do you think R-CASTLE can foster more research studies of this kind in education?

**FIGURE 12:**
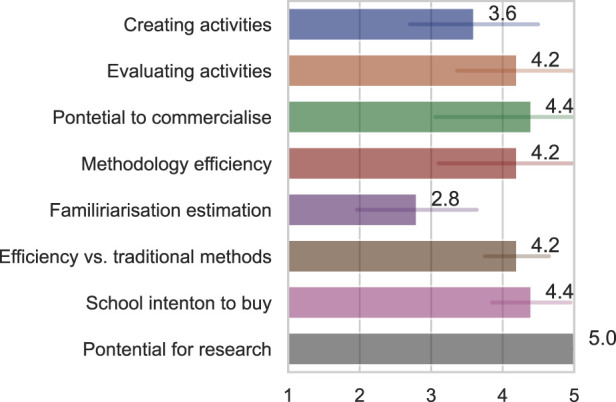
Teachers' perceptions of the architecture interface.

The results, illustrated in Figure 12, showed that teachers have positive attitudes toward all the asked aspects, including the time to familiarise, where the lower the score (low time to familiarise), the better. The point where teachers hesitated the most was related to creating an activity, with an average score of 3.6. They verbalised that they believed they would have difficulties understanding how the windows work at first, but within a few days of using it, they would be able to take full advantage of the system.

The participant teachers possessed a high understanding of the contributions of the proposed architecture to the research in their classrooms, where all the teachers gave a maximum (5) score to this question.

##### 5.3.3.1 What makes a teacher–researcher collaboration successful?

The last question in the item led to the final discussion with the teachers. After giving their answer to the point related to the research question, we further investigated their opinion about it by asking: “what are the important points that make research in SARs efficient in their classroom and how to foster them?”

The key point to the teachers was presenting efficient communication with respect to the goals and benefits of the research. T1 emphasised: “*Researchers must know the reality [in the classrooms] to understand what can be offered by [their] research.*” T2 added a perspective from the teachers’ side, “ *[…] there must be an interest from our part. A sort of motivation to learn something new.*”

Related to the research artefacts, T5 added: “*A demonstration of method effectiveness is required, as well as showing teachers that the robot is an ally, especially when convincing parents and school directors. It that depended only on myself, I would allow research in my classroom only if I am convinced it will bring a good time for my children.*” She concluded that the automatic reports of the evaluation module are a powerful means of intuitively providing this validation.

### 5.4 Discussion

#### 5.4.1 Teacher feedback

Not surprisingly, the challenges the teachers face in the given southeast Brazilian context are well aligned with the literature. Although financial costs are pointed out as the main issue to consolidate the popularisation of SARs in non-scientific activities, the second and third most critical issues, according to the teachers (Teachers’ lack of technical knowledge and Time for preparing activities) can benefit from the advantages of our solution.

When asked which kind of activities they would use the R-CASTLE, all teachers said they consider themselves able to use the architecture for all their activities. Although long-term experiments to validate this hypothesis are required, this unanimous answer suggests a high level of intuitiveness of our proposal.

Public school teachers claimed that the main barrier is the lack of investment in such technologies. In private schools, the main issue is to convince parents and directors about the efficiency of using SARs. Regarding the differences between using SARs as a regular tool or using them as part of research, the critical points present a small variation between these two conditions, except for, of course, the financial cost. As a solution, the fact that the architecture was developed in modules afforded tests using low-cost social robots. In an experiment (Pinto et al., 2018), the performance of the autonomous methods was kept the same when using state-of-art and low-cost robots. Similarly, users reported enjoyment, and their performance did not present significant variance between conditions. This suggests a valuable alternative to approach the problem of using high-cost SARs by simply adapting the architecture features to other devices with the same performance.

The fact that all the interviewed teachers reported that R-CASTLE could smooth all the critical factors we presented to them leads to a consequent hypothesis that this strategy is a potential solution to minimise issues related to teacher skills when using SARs.

Furthermore, the fact that participants in this final validation raised the same critical points the architecture aims to cover (based on the feedback of teachers who participated in the design phase) before knowing about our proposal shows consistency in the issues being approached. The fast finding of solutions using the architecture’s functionality suggests a high potential for this alternative and fast familiarisation time.

#### 5.4.2 Main takeaways

Very often, researchers underestimate the extra time needed to invest in communication with teachers and school staff while doing experiments with social robots. In contrast with other technologies that are better consolidated, for example, tablets, SARs have the distinction of counting on extra levels of complexity, namely a more extensive hardware, a different outcome in children’s expectation due to their social aspect, *etc.* These particularities can be a point of friction for schools, where administrators need to understand the technology better before being compliant in the use of such technologies either for research studies or as a tool for their teachers. As a more concrete example in this paper, three of five teachers pointed out in this interview that providing the teachers with devoted time to explain these factors and the implications of SARs is key to achieving success in research deployed to classrooms. This clarification speaks not only to outcomes but also to the impacts it may have on teachers’ time, planning, and cognitive load. Although the R-CASTLE itself cannot provide such explanations, the teachers noticed it was a powerful means of encapsulating technical settings and illustrating student and system performances.

Related to the strategy for the final validation, the employed interview scheme of gradually presenting the elements of investigation, being the problems, the methodology, the tools, the main goals of the research, and, finally, the artefact of investigation, have helped the participants better understand the concepts and goals of each question. Similarly, the aggregated information from previous sections has supported teachers in building their own critical thinking and chain of thoughts. Hence, we postulate that exposing participants to this process in the final user validation generated a more formulated and reacher to analyse feedback, compared to outcomes of the validation in the design phase, in which we presented the solution instantaneously.

#### 5.4.3 Limitations

We acknowledge several limitations of this work. First, it was performed with a small sample size of teachers from the same region of Brazil: São Paulo. Therefore, the discussions were based on the school practices in this area. Additionally, the teachers had no experience with (social) robots and were not exposed physically to the system or the robots, which may have influenced the results. However, time limitations from the teachers and experimenters led the works approaching the same theme being published over the last decades to have similar small samples of participants (Chang et al., 2010; Serholt et al., 2014; Ahmad et al., 2016; Ceha et al., 2022; Tozadore et al., 2023). Then, our focus with this qualitative analysis lies not in making broad generalisations across populations but rather in providing a detailed portrayal of the sociotechnical context being studied. Additionally, logistical constraints prevented us from implementing the robot in classrooms due to teacher availability. Consequently, while teachers have shown strong positive attitudes toward the architecture, the long-term implications of employing the proposed solution remain unexplored and could contradict what they claimed in their first impression.

## 6 Conclusion

R-CASTLE is a system developed for the educational domain. It is a system that students and teachers can interact with in different ways. Students can learn concepts about diverse subjects and do exercises provided by R-CASTLE, interacting with a robot and the system at the necessary difficulty level for students to improve their scores. Teachers can insert exercises that approach the concepts to be learned by the students more easily without having much knowledge about computer issues. This paper investigated how researchers and teachers can use the R-CASTLE in long-term experiments and how and what design decisions in our architecture helped them in human–robot interaction activities.

In its first phase of development, results from the experiments with students helped to understand the importance of building an autonomous robot that can interact with students through the dialogue and vision modules because these characteristics increased users’ perception of the robot. On the other hand, experiments with teachers showed the importance of presenting the goals and results of the interventions at each phase of the study, which is remarkably enhanced through visual support.

We implemented another round of enhancements based on the results and feedback from these experiments. The implementation of the architecture in modules allowed gathering several algorithms for its use, which makes it easier to adapt the architecture to any solution, whereas the R-CASTLE interface provided faster configuration of the algorithm parameters, the content of the activities, the interaction flow, and the activity outcome analyses.

Once these features were implemented in the R-CASTLE, we performed a qualitative analysis with teachers who did not participate in the design phase (first phase) to check consistency. We took the opportunity to explore open issues of socially assistive robotics in education in the specific context where the architecture was developed. Without knowing about our research, teachers reported having difficulties with the same problems that our approach targets. After we explained our goals, they could intuitively link the architecture’s functionalities to their daily tasks. The interface had the approval of all participant teachers, who reported a very high intention to adopt and a low estimation of time to get used to the system. However, large-scale studies are needed to quantitatively assess this proposal’s potential related to the communication enhancement between researchers and teachers, as well as the researcher of social robots, in long-term experiments in real classrooms.

Finally, the system's modularity affords follow-up studies in many directions for applying machine learning algorithms. For instance, the growing number of studies using large language models would benefit from deploying their algorithms in the dialogue module and analysing the different outcomes of autonomous dialogues. Furthermore, although we limited the applications of this architecture to the educational domain, we believe it can be easily adapted and used in other areas in which there are stakeholders involved in the look, for instance, in the healthcare area, where therapists use the robot in multiple sessions with their patients.

## Figures and Tables

**TABLE A1:** Detailed coding scheme used in the thematic analysis of Section 5.

Codes and sub-classes	Description
Robot’s role	
Personal assistant	The robot can support a person verbally or visually, answering their questions when required
Tutor	The robot provides information and instructions to the user
Peer	The robot acts as a colleague at the same level as the user
Robot’s skills	
Emotion	Robot’s capability to display, try to predict, or approach aspects of human emotions
Storytelling	Robot’s capability to tell stories and narratives
Embodiment	Every component related to the physical aspects of the robot
Novelty effect	Robot’s capability of engaging children due to being a novelty in their routine
Generalisation	Robot’s capability to perform different tasks or roles and approach multiple contexts
Scalability	Robot’s capability to perform a given activity numerous times without a decrease in performance due to fatigue; possibility of placing two or more robots to allow multiple students to perform individual activities at the same time
Barriers	
Teacher familiarisation	Elements related to how easily teachers can use a given technology or how much they are afraid of its impact
Access to the technology	Availability of the resource for the teachers, comprising prices, incentives to use, easy-to-find assistance, *etc.*
Competitive attention with teachers	Elements that can compete with the teachers for the students’ attention: For example, an alarm ringing or a social robot
Price	Barriers related to the financial costs of the robots
Technical aspects	Complexity in setting up equipment (SARs, in this case) and dealing with unforeseen issues like malfunctions or damage

## Data Availability

The raw data supporting the conclusions of this article will be made available by the authors, without undue reservation.

## Footnotes

1 Please note that the studies are presented in the chronological order they were conducted rather than the order they were published.

2
https://www.youtube.com/watch?v=GlNj98L1Mrc

3
https://pypi.org/project/PyQt5/

4
https://pypi.org/project/SpeechRecognition/Accessed in December 2023.

5
http://doc.aldebaran.com/2-1/naoqi/audio/altexttospeech-tuto.html Accessed in December 2023.

6
https://pypi.org/project/gTTS/Accessed in December 2023.

7
https://github.com/ageitgey/face_recognition Accessed on December 2023.

8 This experiment was approved under protocol number 72203717.9.0000.5561 by the Brazilian Ethics Board.

9
https://youtu.be/j23qqcDGUrE?si=NxnApWQ_BBTIukt_&t=171

10 A complete description of these factors is presented in Appendix Table A1 in the appendix.
